# Iron Fists or Velvet Gloves? Puberty Stress, Parenting Style, and Social Evaluative Distress Among Chinese Adolescents

**DOI:** 10.3390/bs16060837

**Published:** 2026-05-22

**Authors:** Yongqi Xu, Ruining Jin

**Affiliations:** 1 Institute of Higher Education, Beijing University of Technology, Beijing 100124, China; 2 School of Education, Minzu University of China, Beijing 100081, China; 22400194@muc.edu.cn

**Keywords:** puberty stress, adolescent, parenting style, social evaluative distress

## Abstract

Background: Puberty is a period of visible bodily change, heightened self-consciousness, and increased sensitivity to social evaluation. While prior studies have linked pubertal development to broad psychological outcomes, less attention has been given to adolescents’ social evaluative distress, defined here as discomfort when feeling looked at or talked about by others. Parenting style may also be relevant to this outcome. Methods: Using secondary survey data from 3591 secondary-school students in Shenzhen, China, this study employed Bayesian analysis to examine whether puberty stress, authoritarian parenting, and permissive parenting were associated with adolescents’ social evaluative distress, and whether authoritarian and permissive parenting moderated the association between puberty stress and social evaluative distress. Results: Puberty stress was positively associated with social evaluative distress, and authoritarian parenting was also positively associated with this outcome. Permissive parenting did not show a clear direct association. Neither authoritarian nor permissive parenting showed clear evidence of moderating the association between puberty stress and social evaluative distress. Conclusions: Social evaluative distress during adolescence appears to be associated more clearly with puberty stress and authoritarian parenting as direct correlates than with interaction effects between puberty stress and parenting style. The study extends existing literature by focusing on a narrower, socially focused form of adolescent distress in the Chinese context.

## 1. Introduction

### 1.1. Pubertal Change, Puberty Stress, and Social Evaluative Distress

As a transitional stage between childhood and adulthood, puberty is a time marked by rapid growth and changes among adolescents, including the emergence of secondary sexual features, the attainment of fertility, and significant psychological and identity shifts (Kipke, 1999). Specifically, males during this stage will undergo enlargement of the scrotum and testes, penis enlargement, pubic hair development, voice deepening, facial hair growth, increased muscle mass, broadening of shoulders (Loomba-Albrecht & Styne, 2009), whereas females will have breast development, pubic hair development, growth spurt, onset of menstruation, and widening of hips (Colvin & Abdullatif, 2013). The biological and physical changes are usually coupled by psychological and identity changes, as adolescents during puberty tend to form a higher propensity to seek out new experiences, a heightened awareness of vulnerability, a lowered perception of risks and danger, a stronger desire for independence, and an inward quest for self-identity (Nebhinani & Jain, 2019).

Because these changes are often highly visible and occur unevenly across adolescents, they may be experienced not only as biological development but also as a source of puberty stress. Empirical research has shown that pubertal development, and especially early or off-time maturation, is associated with more negative body-related self-perceptions, greater interpersonal stress, and heightened vulnerability to internalizing difficulties during adolescence (Hamilton et al., 2014; Jiang et al., 2021; Padrutt et al., 2023). These patterns are especially important because puberty unfolds in a social environment. Adolescence is a period of heightened sensitivity to social evaluation, stronger self-consciousness, and greater concern with how one is perceived by others, as research on adolescent socio-affective development suggests that young people become particularly reactive to situations involving judgment, scrutiny, or peer evaluation during this stage (Guyer et al., 2014; Silk et al., 2012; Somerville, 2013).

In light of this developmental context, one plausible consequence of puberty stress is a form of distress centered on perceived social scrutiny. In the present study, this is conceptualized as social evaluative distress, referring to discomfort when one feels looked at or talked about by others. Social evaluative distress is important in its own right. Adolescents with heightened self-consciousness and sensitivity to social evaluation tend to report more internalizing difficulties, and socially evaluative vulnerability has been linked to anxiety, depressive symptoms, and poorer peer functioning (Bowker & Rubin, 2009; Chiu et al., 2021; Silk et al., 2012) Experimental work further suggests that social evaluation can negatively affect adolescents’ mood and cognitive functioning in the moment, underscoring the developmental significance of this form of distress (Grunewald et al., 2022) Prior studies have generally examined broader adjacent outcomes, such as depression, internalizing symptoms, negative physical self, and interpersonal stress, rather than directly testing whether felt puberty stress is associated with the narrower, socially focused outcome examined here, namely social evaluative distress (Jiang et al., 2021; Natsuaki et al., 2009; Somerville, 2013).

### 1.2. Parenting and Adolescents’ Emotional Distress

Parenting remains a central context for adolescents’ emotional development, even as peer relationships become increasingly salient during adolescence. Parenting style has been linked to adolescents’ internalizing symptoms in meta-analytic work (Pinquart, 2017), to emotional and behavioral problems in Chinese adolescents (Wang et al., 2024), and to socially oriented distress such as adolescent social anxiety in both review and empirical research (Gómez-Ortiz et al., 2019; Ilyas & Khan, 2023). In Chinese samples specifically, parenting style has been shown to predict adolescents’ emotional and behavioral problems, while broader reviews have also tied parenting to adolescent depression and social anxiety-related outcomes (Liu & Merritt, 2018; Wang et al., 2024).

Within this broader literature, authoritarian and permissive parenting warrant separate attention because they capture different combinations of parental control and responsiveness and may therefore relate to adolescent distress in different ways (Pinquart, 2017). Meta-analytic evidence shows that parenting styles are differentially associated with children’s and adolescents’ internalizing symptoms rather than exerting uniform effects across styles (Pinquart, 2017). Authoritarian parenting has been linked more consistently to adverse emotional outcomes, including depression and related internalizing symptoms (Liu & Merritt, 2018; Pinquart, 2017). By contrast, the literature on permissive parenting is less consistent but still suggests potential mental health relevance. For example, internalizing problems were greatest among behaviorally inhibited children who were also exposed to permissive parenting in Williams et al.’s study (Williams et al., 2009), and Lo et al. reported that permissive parenting may contribute to childhood anxiety when insufficient guidance and direction are provided (Lo et al., 2020). A recent review likewise noted that findings on permissive parenting and depression/anxiety vary across studies, with some reporting significant associations and others reporting no clear relationship (Yilmaz & Enez Darçin, 2025).

### 1.3. Authoritarian and Permissive Parenting in the Chinese Context

On the sociohistorical front, Chinese culture is deeply influenced by Confucianism—a philosophical and ethical system that places great emphasis on collectivist principles (Huang & Gove, 2012). These principles include adherence to societal norms, conformity to authority, cultivation of strong interpersonal connections, and a preference for conflict avoidance for the sake of group harmony (Peterson et al., 2005). Within this rigid hierarchical structure, there is a discouragement of individuals’ desires for autonomy and any actions that may pose a risk to group cohesion, as parents usually use various control approaches, including psychological control (guilt induction, shame and love withdrawal) to reinforce parental authority and promote prosocial behaviors (Fuligni, 1998; Lau, 1992; Le & Jin, 2024). Consequently, the authoritarian parenting style is the traditional parenting style that is commonly upheld by many Chinese parents. On the sociopolitical front, China has distinguished itself globally through the implementation of the controversial “One Child Policy,” which was enforced by the government from 1979 to 2016. This policy led to a “4-2-1” family configuration, which consists of four grandparents, two parents, and one child (Bi et al., 2018). In such a family structure, many grandparents tend to pamper their only child (Bi et al., 2018), and parents also adapted their parenting approach in response to the policy, becoming more child-centered and relaxed on discipline (Chuang & Su, 2009). These developments do not imply a uniform transformation of Chinese parenting, but they do suggest that more permissive tendencies may coexist with more traditional authoritarian practices in some family settings.

Taken together, these sociohistorical and sociopolitical dynamics suggest that both authoritarian and permissive parenting are meaningful styles to examine in Chinese adolescents, as they reflect two distinct yet contextually plausible family environments. Thus, examining both styles helps situate adolescent emotional distress within a Chinese family context.

### 1.4. Theoretical Frameworks

Two theoretical perspectives help explain why puberty stress may be associated with adolescents’ social evaluative distress and why parenting may also matter in this process. The first is Social Comparison Theory (SCT), originally proposed by Festinger (Festinger, 1954), which suggests that individuals have a basic tendency to evaluate themselves by comparing their attributes, abilities, and experiences with those of others. Social comparison is especially relevant when objective standards are unclear and when individuals are uncertain about how to interpret personal change or difference (Festinger, 1954). During puberty, adolescents undergo highly visible and uneven bodily development, making peer comparison particularly salient. Because adolescents become increasingly sensitive to peer judgment and social evaluation during this stage, pubertal change may be interpreted not only as a physical process but also as a socially meaningful one (Guyer et al., 2014; Somerville, 2013). Under such conditions, adolescents who experience puberty as stressful may become more vulnerable to discomfort under perceived social scrutiny, especially when their development appears different from what they observe in peers or what they believe others expect. In this sense, SCT helps explain why puberty stress may be linked to social evaluative distress, that is, distress centered on feeling looked at, judged, or talked about by others.

The second perspective is Self-Determination Theory (SDT), particularly its emphasis on the basic psychological needs for autonomy, competence, and relatedness (Ryan & Deci, 2000). SDT argues that psychological well-being is supported when these needs are adequately met and undermined when they are persistently frustrated. This framework is useful for understanding why parenting may be relevant to adolescents’ socially focused distress. A more authoritarian parenting environment, characterized by stronger control and lower responsiveness, may frustrate adolescents’ needs for autonomy and relatedness by limiting independent self-expression and reducing feelings of emotional security. By contrast, permissive parenting may offer warmth but insufficient structure, which may weaken adolescents’ sense of competence in managing stress and navigating socially challenging situations. From an SDT perspective, these two parenting styles may therefore relate to adolescent distress through different mechanisms rather than identical ones. Applied to the present study, SDT provides a conceptual rationale for examining whether authoritarian and permissive parenting are associated with adolescents’ social evaluative distress and whether they may shape how puberty stress is experienced in the social domain.

### 1.5. Current Study

Despite growing research on pubertal development, adolescent distress, and parenting style, prior studies have more often examined broader outcomes such as depression, internalizing symptoms, interpersonal stress, and social anxiety than the narrower, socially focused outcome examined here. In addition, although parenting has been linked to adolescent emotional difficulties, less attention has been paid to whether different parenting styles are associated with adolescents’ social evaluative distress. Addressing this gap is important because adolescence is a developmental period in which bodily change, peer comparison, and family interaction may jointly shape how young people experience social scrutiny and emotional discomfort.

Against this background, the present study examines the associations among puberty stress, authoritarian parenting, permissive parenting, and social evaluative distress in a Chinese context. The primary focus is to investigate whether puberty stress and the two measured parenting styles are associated with adolescents’ social evaluative distress. In addition, the study further explores whether authoritarian and permissive parenting may serve as moderating factors in the association between puberty stress and social evaluative distress. Accordingly, the study addresses the following research questions:

RQ1. To what extent, if any, are puberty stress, authoritarian parenting, and permissive parenting associated with adolescents’ social evaluative distress?
RQ2. To what extent, if any, do authoritarian parenting and permissive parenting moderate the association between puberty stress and adolescents’ social evaluative distress?

## 2. Method

### 2.1. Data

The current study used a secondary dataset describing Chinese secondary school students’ stressors. The dataset was retrieved from the open data article “Data for teenagers’ stressor, mental health, coping style, social support, parenting style and Self-efficacy in South China” (Chen et al., 2020). The data collection by Chen et al. (2020) was approved and supported by the Projects of Philosophy and Social Sciences Research, Ministry of Education of China (Grant No. 18YJC190013).

### 2.2. Sampling Size and Sampling Technique

The dataset reported in the original article was obtained from a sample of 3784 secondary-school students in Shenzhen, Guangdong Province. The sample included 1987 boys and 1797 girls, with a mean age of 14.6 years (SD=1.82). Participants were recruited through a random cluster sampling procedure. Fifteen secondary schools participated in the study, and three classes were randomly selected from each grade level (Grades 7, 8, 10, and 11). Because some cases contained missing values on one or more focal variables, only complete responses were retained for the present analysis. After excluding incomplete cases, the final analytic sample consisted of 3591 participants, indicating that 193 cases (5.1% of the original sample) were removed due to missing data.

Table 1 shows the variables extracted from the original dataset that are used for analytical model constructions.

### 2.3. Model Construction

To probe the possible association between (1) puberty stress, authoritarian parenting, permissive parenting, and social evaluative distress, and (2) the possible moderating impacts of authoritarian and permissive parenting on the association between puberty stress and social evaluative distress, the research model was constructed as follows.

(1) $$Evadistress\;\sim \;normal\left(\mu ,\sigma \right)$$

(2) $$\mu_{i}=\beta_{0}+\beta_{PubertyStress}\times {PubertyStress}_{i}+\beta_{Authoritarian}\times {Authoritarian}_{i}\;{+\;\beta_{Permissive}\times \;{Permissive}_{i}+\beta }_{PubertyStress*Authoritarian}\times {PubertyStress}_{i}\times {Authoritarian}_{i}+\beta_{PubertyStress*Permissive}\times \;{PubertyStress}_{i}\times {Permissive}_{i}$$

Regarding the outcome variable Evadistress, $\mu_{i}$ measures student i’s expected level of social evaluative distress, operationalized as the degree of discomfort when feeling looked at or talked about by others through participants’ agreement to the question “Feel uncomfortable when others look at you or talk about you”. Student i’s perceived stress because of physical changes during puberty is ${PubertyStress}_{i}$. Puberty stress was operationalized using the item “Physical changes (such as adolescence)” from the Stressors Scale for Middle School Students (SSMSS) (Zheng & Chen, 1999). Student i’s perceived level of authoritarian parenting style is ${Authoritarian}_{i}$. Authoritarian parenting was operationalized using a single extracted item, “My parents have strict restrictions on what I should do and what I should not do.” This item taps the strict control/demandingness dimension commonly associated with authoritarian parenting (Kuppens & Ceulemans, 2019; Li et al., 2023), although it does not capture the full breadth of the construct, particularly low warmth or responsiveness. Student i’s perceived level of permissive parenting style is ${Permissive}_{i}$. Permissive parenting was operationalized using the item, “My parents can let me develop naturally.” This item was used as a single-item indicator of a low-control, non-intrusive parenting tendency, which is conceptually closer to the permissive end of the parenting-style continuum than to more controlling styles. At the same time, it should be understood as capturing a narrow aspect of permissive parenting rather than the full construct, which is typically defined by low demandingness together with relatively high responsiveness (Kuppens & Ceulemans, 2019; Li et al., 2023). The model has an intercept $\beta_{0}$ and coefficients $\beta_{PubertyStress}$, $\beta_{Authoritarian},\;\beta_{Permissive},\;\beta_{PubertyStress*Permissive}$ and $\beta_{PubertyStress*Authoritarian}$.

### 2.4. Analysis and Validation

The present study employed a Bayesian analytical approach estimated via Markov Chain Monte Carlo (MCMC) sampling. In this context, MCMC was used to approximate the posterior distributions of the model parameters rather than to transform the observed data themselves (McElreath, 2020). A Bayesian framework was adopted because it allows parameter uncertainty to be expressed probabilistically and supports interpretation through posterior estimates and 95% credible intervals (Wagenmakers et al., 2018).

The model was specified with a Gaussian likelihood for the outcome variable. Because the focal outcome was measured on a 5-category ordinal scale, the Gaussian specification should be understood as a pragmatic approximation; this modeling choice is addressed further in the Limitations section.

Model convergence and sampling efficiency were assessed using multiple standard diagnostics. These included visual inspection of trace plots, the Gelman–Rubin shrink factor (Rhat), and the effective sample size (*n_eff*). Values of Rhat close to 1.00 and sufficiently large *n_eff* (n_eff larger than 1000) were taken as evidence of satisfactory chain convergence and sampling efficiency (Brooks & Gelman, 1998; McElreath, 2020).

To evaluate the reliability of predictive approximation, the study used Pareto-smoothed importance sampling leave-one-out cross-validation (PSIS-LOO) (Vehtari et al., 2017; Vehtari & Gabry, 2019). In this framework, the Pareto-k values were used to assess whether the leave-one-out approximation was stable; lower values (k values lower than 0.5) indicate a more reliable approximation, whereas higher values (k values larger than 0.7) suggest that some observations may exert disproportionate influence on the approximation (Vehtari et al., 2017). Accordingly, PSIS-LOO was used as a predictive diagnostic rather than as a general proof of model fit.

The analyses were performed using the bayesvl open package in R (Version 0.8) (La & Vuong, 2019). We employed uninformative priors to reduce subjectivity’s impact on the estimation. The MCMC setup includes 5000 iterations with 2000 iterations for warm-up and 4 chains. The procedure, involving variable selection to model construction, analysis, validation, and presentation of results, followed the protocols for MCMC-aided Bayesian analysis (Vuong et al., 2022).

## 3. Results

**Figure 1:**
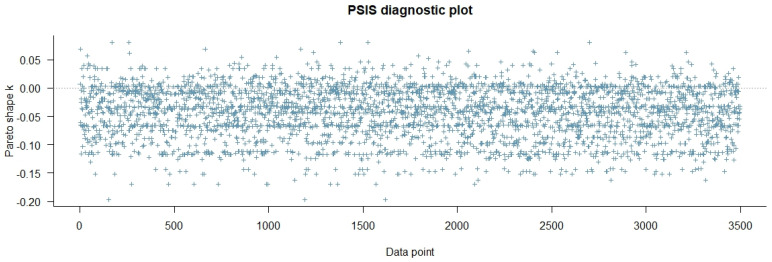
PSIS diagnostic plot.

The PSIS-LOO diagnostics indicated a stable leave-one-out predictive approximation, with all Pareto-k values below 0.5 (see Figure 1). This suggests that the PSIS-LOO approximation was reliable for the present model and that no observations exerted disproportionate influence on the leave-one-out estimates.

As shown in Table 2, all parameters had effective sample sizes greater than 1000 and Rhat=1.00, indicating satisfactory chain convergence and sampling efficiency.

**Figure 2:**
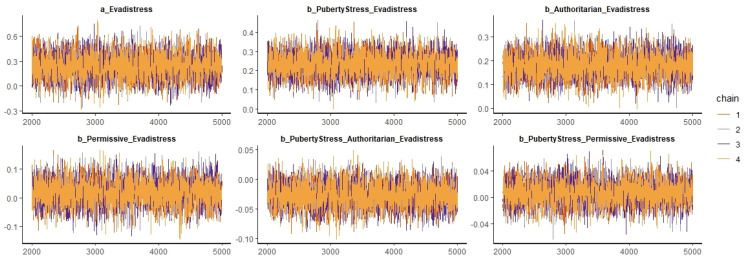
The model’s trace plots.

**Figure 3:**
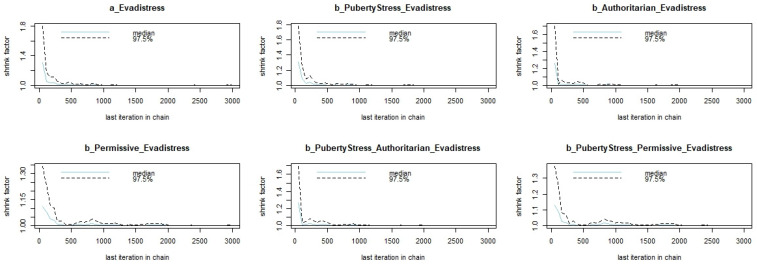
The model’s Gelman–Rubin–Brooks plots.

**Figure 4:**
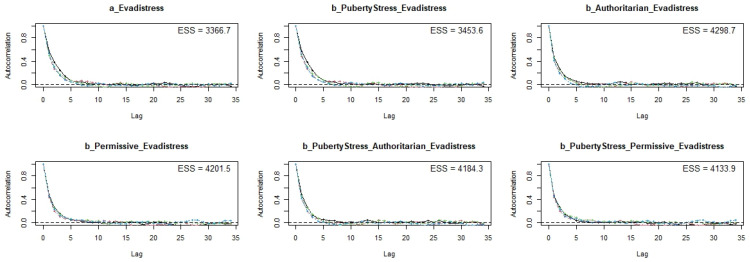
The model’s autocorrelation plots.

The trace plots for the parameters show good convergence of the Markov chains (see Figure 2). The Gelman–Rubin–Brooks plots (See Figure 3) indicate that *Rhat* values drop quickly to 1 in the warm-up period. The autocorrelation plots (See Figure 4) also suggest a quick elimination of problematic autocorrelation among simulated data points within the MCMC processes.

**Figure 5:**
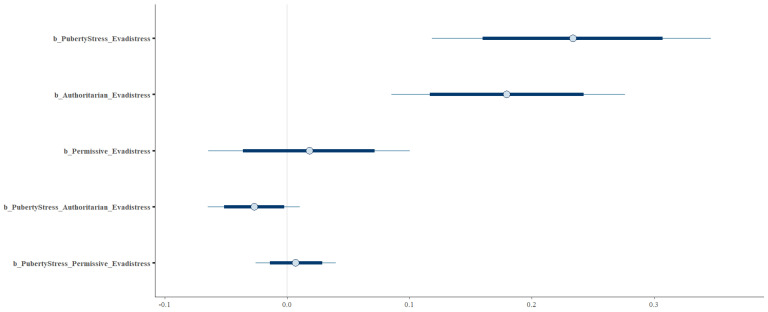
Model’s posterior distributions.

According to the results shown in Table 2, *PubertyStress* was positively associated with social evaluative distress, *Evadistress* $\left(M=0.23,\;SD=0.06\right)$, and Authoritarian parenting, *Authoritarian*, was likewise positively associated with the outcome $\left(M=0.18,\;SD=0.05\right)$. The coefficient for permissive parenting, *Permissive*, was small $\left(M=0.02,\;SD=0.04\right)$, and the posterior interval overlapped zero, suggesting no clear association with social evaluative distress. For the interaction terms, *PubertyStress* × *Authoritarian* showed a small negative coefficient $\left(M=-0.03,\;SD=0.02\right)$, whereas *PubertyStress* × *Permissive* remained close to zero $\left(M=0.01,\;SD=0.02\right)$. Figure 5 further illustrates that the posterior intervals for *PubertyStress* and *Authoritarian* were clearly located on the positive side, whereas the posterior intervals for *Permissive* and both interaction terms crossed zero. Taken together, these findings suggest that puberty stress and authoritarian parenting were directly associated with greater social evaluative distress, but the model did not provide clear evidence on permissive parenting’s association with social evaluative distress. Likewise, the model did not provide clear evidence that authoritarian or permissive parenting moderated the association between puberty stress and social evaluative distress.

## 4. Discussion

Through a Bayesian analysis aided by MCMC sampling on data from 3591 secondary-school students in Shenzhen, China, the present study suggests that puberty stress was positively associated with adolescents’ social evaluative distress, and authoritarian parenting was also positively associated with this outcome. By contrast, permissive parenting did not provide clear evidence of a direct association with social evaluative distress, and neither authoritarian nor permissive parenting showed clear evidence of moderating the association between puberty stress and social evaluative distress.

The positive association between puberty stress and social evaluative distress is consistent with prior literature showing that pubertal development, particularly when experienced as stressful, early, or off-time, is associated with negative physical self-perceptions, interpersonal stress, and internalizing vulnerability during adolescence (Hamilton et al., 2014; Jiang et al., 2021; Natsuaki et al., 2009). This pattern is also theoretically consistent with SCT, which suggests that individuals evaluate themselves partly through comparison with others when objective standards are uncertain (Festinger, 1954; Van Lange, 2008). During puberty, visible and uneven bodily changes could make peer comparison especially salient, while adolescence is also a period of heightened sensitivity to social evaluation and peer judgment (Guyer et al., 2014; Silk et al., 2012; Somerville, 2013). Under such conditions, adolescents who experience puberty as stressful may become more vulnerable to discomfort under perceived social scrutiny, including distress when they feel looked at or talked about by others. The present findings therefore suggest that puberty stress may matter not only through broad internalizing pathways, but also through a narrower socially evaluative route.

The positive association between authoritarian parenting and social evaluative distress is likewise meaningful. Prior research has linked authoritarian parenting to depression, internalizing symptoms, and broader emotional difficulties among children and adolescents (Liu & Merritt, 2018; Xu & Ren, 2025). In the present context, this pattern may be understood as consistent with the possibility that more controlling and less responsive family environments are associated with greater sensitivity to criticism, scrutiny, and external judgment. From the SDT perspective, such a family environment may frustrate adolescents’ needs for autonomy and relatedness by limiting independent self-expression and reducing feelings of emotional security (Ryan & Deci, 2000). For adolescents already navigating visible bodily change and heightened self-consciousness during puberty, a more controlling and less responsive parenting environment may therefore be associated with greater discomfort in socially evaluative situations. Such a conclusion has been corroborated in recent studies (Fu et al., 2025; Howard et al., 2025; James et al., 2026).

By contrast, permissive parenting did not show a clear direct association with social evaluative distress. This finding is interesting and also understandable in light of the existing literature. Compared with authoritarian parenting, the evidence on permissive parenting and youth mental health has generally been more mixed and less consistent (Pinquart, 2017; Yilmaz & Enez Darçin, 2025). One possible interpretation is that permissive parenting may be less directly tied to adolescents’ discomfort under perceived social scrutiny than authoritarian parenting is. In the context of Shenzhen, an economically affluent megacity where younger generations have been increasingly shaped by processes of individualization (Yan, 2020), the relative autonomy associated with permissive parenting may not necessarily intensify socially evaluative distress. Although such parenting may provide less guidance and intervention, it may also leave greater room for adolescents to draw on their own agency when navigating socially evaluative situations. Under these conditions, social evaluative distress may depend more on adolescents’ individual coping resources and interpersonal competence, which may partly account for the unclear association observed in the present study.

Another important result is that neither authoritarian nor permissive parenting showed clear evidence of moderating the association between puberty stress and social evaluative distress. This suggests that the relationship between puberty stress and socially focused distress may be better understood as relatively additive than interactive in this sample. One possible interpretation is that parenting style functions more as a background relational climate than as a contingent amplifier of puberty-related stress. Under this reading, authoritarian parenting may elevate adolescents’ overall vulnerability to socially focused distress, but not necessarily intensify the specific link between puberty stress and that distress. This interpretation is also consistent with the broader methodological point that interaction effects are often smaller and harder to detect than direct associations.

## 5. Implications

The findings have several implications for research and practice. First, they suggest that puberty stress is relevant not only to broad internalizing difficulties, but also to a narrower and more socially focused form of distress centered on perceived scrutiny from others. This extends prior research that has more often emphasized depression, anxiety, negative physical self, or interpersonal stress by showing that adolescents’ discomfort when feeling looked at or talked about may also be meaningfully associated with pubertal stress. In this respect, the study highlights the value of examining more specific forms of adolescent distress rather than relying only on broad psychological outcomes.

Second, the findings indicate that authoritarian parenting may be more important as a direct correlate of adolescents’ social evaluative distress than as a contextual amplifier of puberty stress. This distinction matters because it suggests that a more controlling and less responsive parenting environment may be associated with adolescents’ socially focused distress even in the absence of a strong moderation pattern. In practical terms, this points to the importance of family environments that reduce excessive control, criticism, and rigid behavioral demands during adolescence, particularly when young people are already navigating visible bodily change and heightened self-consciousness. Parenting guidance and school-based family education may therefore benefit from addressing not only adolescents’ physical and emotional adjustment during puberty, but also the relational climate in which that adjustment takes place.

Third, the lack of clear evidence for permissive parenting and for the two moderation effects is also informative. These findings suggest that different parenting styles should not be assumed to influence adolescent distress in identical ways, and that direct associations may be more robust than conditional interaction effects in this context. This has methodological as well as substantive value. It indicates that future studies should distinguish carefully between asking whether parenting is associated with adolescent distress at all and asking whether parenting changes the strength of other developmental associations. In the present case, the evidence supports the former more clearly than the latter.

Finally, the findings also have contextual significance for research on Chinese adolescents. Existing studies in China have often linked parenting to broader emotional and behavioral outcomes, but less attention has been given to socially focused distress arising under perceived scrutiny. By examining puberty stress, parenting, and social evaluative distress together, the study contributes to a more differentiated understanding of how developmental stress and family context may operate within a Chinese urban setting.

## 6. Limitations

Several limitations should be acknowledged. First, the study relied on a cross-sectional secondary dataset, which means that the observed associations should not be interpreted causally. Although the theoretical framing suggests plausible pathways linking puberty stress and parenting style to social evaluative distress, the design does not permit conclusions about temporal ordering or causal direction. Future research would benefit from longitudinal data that can better capture how pubertal stress, family interaction, and socially focused distress unfold over time.

Second, the focal variables were operationalized using single extracted items from the secondary dataset rather than validated multi-item scales designed specifically for the present study. This is an important measurement limitation. Social evaluative distress was measured through a single item referring to discomfort when others look at or talk about the adolescent, puberty stress was measured through a single item on physical changes during puberty, and authoritarian and permissive parenting were each represented by single items capturing relatively narrow dimensions of those parenting styles. As a result, internal consistency reliability could not be assessed, and the measures may contain greater measurement error than fuller multi-item scales. Moreover, complex constructs such as authoritarian and permissive parenting involve multiple dimensions, including demandingness, responsiveness, control, warmth, and autonomy support, which cannot be fully represented by one item. Although the selected items are conceptually relevant to the constructs examined here, the findings should therefore be interpreted as applying to these specific item-level indicators or single-item proxies, rather than to the broader constructs in their entirety. Future studies should replicate the analysis using validated multi-item measures of puberty-related stress, parenting style, and social evaluative distress.

Third, the analytical model treated the outcome as a Gaussian variable even though it was measured on a 5-category ordinal scale. This specification was used as a pragmatic approximation, but it remains a limitation of the study. Future research could strengthen the analysis by applying ordinal Bayesian models and by using fuller multi-item measures that better capture the breadth of puberty-related stress, parenting style, and socially evaluative distress.

Fourth, the final analytic sample was based on complete-case deletion after excluding participants with missing values on one or more focal variables. Although the proportion of excluded cases was relatively modest, complete-case analysis may introduce bias if the missingness was systematic rather than random. In addition, no formal sensitivity analysis was conducted to test whether the substantive findings would remain stable under alternative missing-data strategies, such as multiple imputation. The results should therefore be interpreted with appropriate caution.

Fifth, the dataset was obtained through cluster sampling across schools and classes, and the analysis did not explicitly model potential clustering effects at the school or class level. In addition, the model did not incorporate a wider set of possible covariates beyond the focal variables. Factors such as age, grade, sex, peer context, or other family characteristics may also be relevant to adolescents’ social evaluative distress. Future studies could build on the present findings by using more fully specified models that incorporate multilevel structure and a broader range of covariates.

Finally, the study was based on adolescents from Shenzhen, a rapidly modernized and highly competitive urban context in South China. Although this setting is substantively important, the findings may not generalize directly to adolescents in rural areas, other Chinese regions, or non-Chinese settings. Further research across more diverse social and cultural contexts would be useful for assessing the broader applicability of the observed patterns.

## Figures and Tables

**Table 1:** Variable description.

Variable	Description	Value
PubertyStress	The level of felt stress due to physical changes during puberty	0. no effect 1. slight effect 2. moderate effect 3. severe effect 4. extremely severe effect
Evadistress	The participant’s degree of uncomfortable feeling when others look at them or talk about them	0. never 1. slight 2. moderate 3. heavy 4. serious
Permissive	The participant’s perceived degree of permissive parenting	1. never 2. occasionally 3. often 4. always
Authoritarian	The participant’s perceived degree of authoritarian parenting	1. never 2. occasionally 3. often 4. always

**Table 2:** Simulated posteriors.

Parameters	Mean (M)	Standard Deviation (S)	n_eff	Rhat
Constant	0.24	0.14	3317	1
PubertyStress	0.23	0.06	3326	1
Authoritarian	0.18	0.05	4359	1
Permissive	0.02	0.04	3700	1
PubertyStress × Authoritarian	−0.03	0.02	4345	1
PubertyStress × Permissive	0.01	0.02	3585	1

## Data Availability

The dataset used in this study can be accessed at https://plu.mx/plum/a/?doi=10.1016/j.dib.2020.105202 (accessed on 25 March 2026).
